# Individualized Endoscopic Surveillance for Metachronous Gastric Cancer After Endoscopic Submucosal Dissection: A Retrospective Observational Study

**DOI:** 10.5152/tjg.2023.22655

**Published:** 2023-07-01

**Authors:** Keting Huang, Duochen Jin, Guoxin Zhang

**Affiliations:** Department of Gastroenterology, First Affiliated Hospital of Nanjing Medical University, Nanjing, China

**Keywords:** Metachronous gastric cancer, Endoscopic surveillance, Endoscopic submucosal dissection, Early gastric cancer, High-grade dysplasia

## Abstract

**Background/Aims:**

Endoscopic submucosal dissection has been widely applied for curative resection of early gastric cancer or high-grade dysplasia, and metachronous gastric cancer is a major issue after endoscopic therapy. Here, we studied the recurrence patterns of metachronous gastric cancer and its correlation with the primary lesions.

**Materials and Methods:**

A total of 286 consecutive patients undergoing endoscopic submucosal dissection for early gastric cancer or high-grade dysplasia between March 2011 and March 2018 were retrospectively reviewed. Metachronous gastric cancer was defined as a new gastric cancer detected more than 1 year after endoscopic submucosal dissection.

**Results:**

During a median follow-up of 36 months, 24 patients developed metachronous gastric cancer. The 5-year cumulative incidence was 13.4% and the annual incidence was 24.3 cases per 1000 person-years. Subgroup analysis revealed that the third year after early gastric cancer resection and the fifth year after high-grade dysplasia resection were the predilection periods of metachronous gastric cancer. Correlation analysis suggested that the metachronous and primary lesions showed a significant correlation in cross-sectional position (C = 0.627, *P* = .027) but not in pathological characteristics (*P* > .05). When the primary lesions were located in the posterior walls, the metachronous lesions were prone to occur in the lesser curvatures (C = 0.494, *P* = .008) and the reverse was also true (C = 0.422, *P* = .029).

**Conclusion:**

The predilection periods and common sites of metachronous gastric cancer are associated with the primary lesions. Meticulous individualized endoscopic surveillance after endoscopic submucosal dissection requires to be conducted, taking into account the characteristics of primary lesions.

**Main Points**

It was the first study to investigate the temporal-spatial association between metachronous and primary gastric cancer.
The third year after early gastric cancer resection and the fifth year after high-grade dysplasia resection were the predilection periods of metachronous gastric cancer.
The metachronous and primary lesions shared close locations, but not similar pathological characteristics.

## INTRODUCTION

Early gastric cancer (EGC) is a malignant tumor restricted to the gastric mucosa and submucosa, with a 5-year survival rate of 90% or more.^1^ Endoscopic submucosal dissection (ESD) has been recommended as a first-line treatment modality for EGC.^2^ Although ESD delivers a survival rate comparable to that of gastrectomy, the development of metachronous gastric cancer (MGC) in the remnant mucosa is a major concern requiring careful consideration.^3^

Metachronous gastric cancer is a de novo gastric cancer that develops more than 1 year after the curative ESD.^3^ The disease-specific survival of MGC patients at 5 and 10 years were 99.2% and 92.5%, respectively.^4^ It was reported that under scheduled endoscopic surveillance, the incidence of invading gastric cancer after ESD was extremely low at only 0.12%.^5^ Thus, scheduled endoscopic follow-up after ESD is indispensable to early diagnosis and treatment of MGC.

Effective endoscopic surveillance should be based on in-depth knowledge of MGC. Previous studies have shown that the incidence of MGC after ESD ranges from 3.5% to 15.6%.^4,6,7^ High-grade dysplasia (HGD), with a malignant tendency to progress to invasive gastric cancer in a short time, is also recommended as an indication for ESD. Cumulative incidence of MGC after HGD resection was comparable to that after EGC resection.^8^ *Helicobacter pylori *(*H. pylori*) infection and precancerous lesions, such as atrophy and intestinal metaplasia, are often considered risk factors for MGC.^9,10^ However, the knowledge regarding recurrence patterns of MGC was not meticulous, ignoring individual differences. In addition, whether the metachronous lesions are associated with the primary lesions that could improve the early detection efficiency of MGC has not been evaluated.

In this study, we retrospectively analyzed the recurrence patterns of MGC after ESD, including predilection period, susceptible population, and common sites, and evaluated potential associations between the metachronous and primary lesions. We aimed to provide new ideas for effective endoscopic surveillance after ESD treatment.

## MATERIALS AND METHODS

### Study Design and Patients

This retrospective cohort study included patients who underwent ESD at the First Affiliated Hospital of Nanjing Medical University, Nanjing, China from March 2011 to March 2018. The indications for ESD and the definition of curative resection followed the guidelines of the Japanese Gastric Cancer Association.^2^ Patients with complete clinical data were eligible if they underwent curative resection and their postoperative histopathology was confirmed as EGC or HGD. Patients with the following conditions were excluded: (i) a history of gastrectomy; (ii) additional gastrectomy, radiotherapy, or chemotherapy within 1 year; and (iii) a follow-up of less than 1 year. This study was approved by the ethics committee of the First Affiliated Hospital of Nanjing Medical University (No. 2018-SR-211) and followed the principles of the Helsinki Declaration. Informed consent was obtained from all of the included patients.

### Histopathological Evaluation

The ESD procedures were conducted routinely, and the resected specimens were fixed overnight in 10% formalin solution, followed by embedding, sectioning at 2-mm intervals, and histopathological staining. Two experienced pathologists evaluated the tumor histological types in accordance with the World Health Organization classification. Pathological diagnoses were based on the Vienna classification: HGD and carcinoma in situ were considered to be HGD (categories 4.1 to 4.2), and intramucosal and submucosal carcinoma were considered to be EGC (categories 4.3 to 5).^11^ Macroscopic types were categorized into 3 groups: elevated (type 0-I, IIa), flat (0-IIb), and depressed (type 0-IIc, III).^12^ The tumor location was divided into 3 longitudinal parts, the upper third, middle third, and lower third, and 4 cross-sectional parts, the greater curvature, lesser curvature, anterior wall, and posterior wall.^13^ Gastric mucosal atrophy or intestinal metaplasia was recorded as positive when a histological biopsy from the antrum and body indicated moderate-to-severe atrophy or intestinal metaplasia.^14^

### Detection of *H*. *pylori* and Follow-Up Surveillance

Before ESD, the presence or absence of *H. pylori* infection was detected with the ^13^C urea breath test (Otsuka, Tokushima, Japan) and the rapid urease test. If either of the tests was positive,* H. pylori* infection was deemed to be present. Otherwise, *H. pylori* infection was considered negative. Patients with confirmed infection were recommended to undergo classical bismuth-containing quadruple therapy twice daily for 2 weeks. If the first eradication treatment failed, patients received second-line eradication therapy. Successful eradication was determined with a negative urea breath test 4 weeks or more after eradication treatment. Endoscopy and abdominal computed tomography were performed at 3, 6, and 12 months and annually thereafter to detect residual lesions, recurrence, and metastasis. The follow-up duration was calculated as the interval from the date of index ESD to the date of MGC occurrence or last endoscopic examination before January 20, 2020. The MGC was defined as a newly developed cancer occurring at another site in the stomach at least 1 year after the primary resection.^3,9^

### Statistical Analysis

Continuous variables were expressed as the means ± standard deviations or medians with ranges, and categorical variables were expressed as quantities and frequencies. Intergroup differences were compared with Student’s *t*-test, the chi-square test or Fisher’s exact test. Variables were examined in Cox model to estimate hazard ratios (HRs) and 95% confidence intervals (CIs). All variables with* P *value < .100 in the univariate analysis were subjected to multivariate analysis to identify independent risk factors for MGC. The cumulative incidence of MGC was estimated with Kaplan–Meier methods and compared by the log-rank test. The annual incidence was denoted by person-years and stratified based on the histopathology of primary tumors. Additionally, contingency correlation analysis was utilized to determine associations of the metachronous lesions with the primary lesions. Statistical analyses were performed with IBM Statistical Package for the Social Sciences version 22 software (IBM Corp.; Armonk, NY, USA), and *P *< .05 was deemed to be statistically significant.

## RESULTS

### Patient Characteristics

We retrieved the electronic medical records of 477 patients who underwent ESD for EGC or HGD from March 2011 to March 2018. We excluded patients with non-curative resection (n = 112), incomplete data (n = 2), residual stomach (n = 6), additional gastrectomy and chemotherapy within 1 year (n = 23), and a history of other cancers (n = 3). During the follow-up period, 45 patients lost to follow-up within 1 year were also excluded. As a result, a total of 286 patients were enrolled in the final study, including 135 patients with EGC and 151 patients with HGD. Among them, 71.3% (204/286) of the patients were male, with a mean age of 62.5 ± 9.3 years.

### Incidence of Metachronous Gastric Cancer After Endoscopic Submucosal Dissection

**Figure 1.**
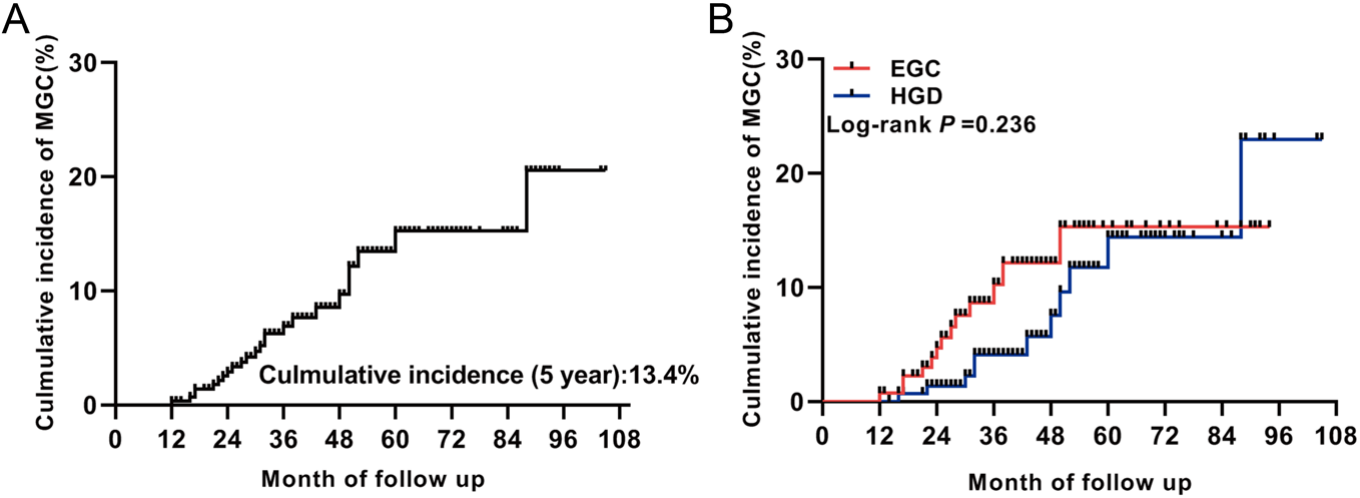
Cumulative incidence curve for MGC after curative ESD. (A) Cumulative incidence of MGC in the whole group. (B) Cumulative incidence of MGC in patients with primary EGC and primary HGD. EGC, early gastric cancer; HGD, high-grade dysplasia; MGC, metachronous gastric cancer.

**Figure 2.**
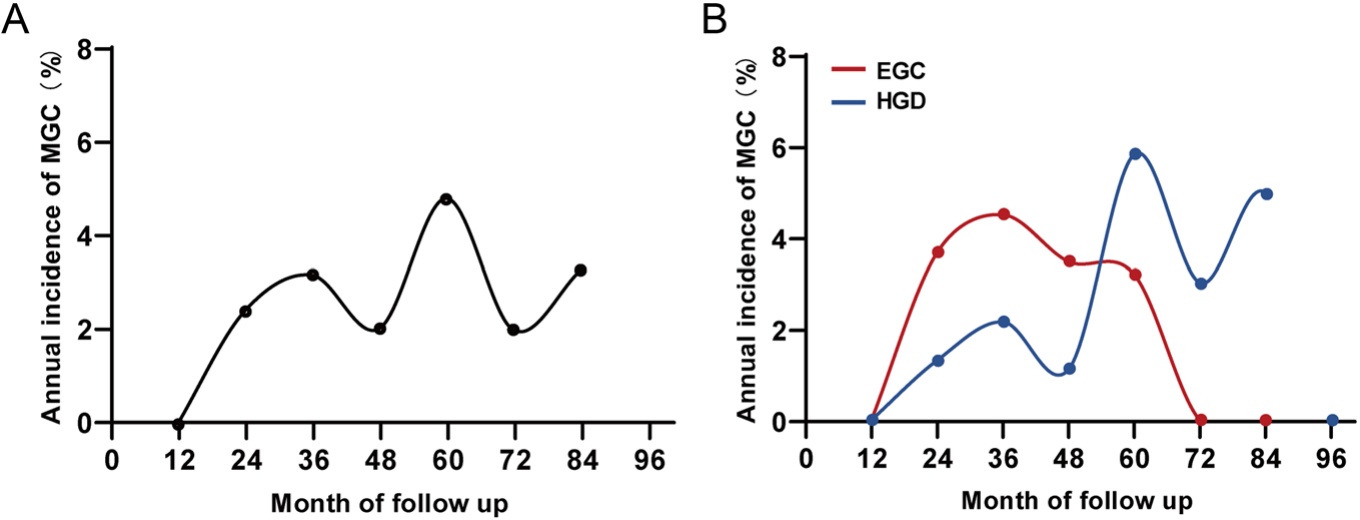
Annual incidence of MGC after curative ESD. (A) Annual incidence of MGC in the whole group. (B) Annual incidence of MGC in patients with primary EGC and primary HGD. EGC, early gastric cancer; HGD, high-grade dysplasia; MGC, metachronous gastric cancer.

During the median follow-up of 36 months (range, 12 to 105 months), MGC developed in 24 patients (24/286, 8.4%). The 5-year cumulative incidence was 13.4%, and the annual incidence was 2.43% (24.3 cases per 1000 person-years) (Figures 1 and 2). Given the differences between primary EGC and HGD in pathogenicity and invasiveness, we stratified the MGC incidence according to the histopathology of primary tumors. There was no significant difference in cumulative incidence between the 2 groups (*P* = .236, log-rank test, Figure 1B), but interestingly, the average time to MGC after endoscopic resection was different (HGD: 43.0 ± 20.1 vs. EGC: 26.9 ± 10.2, *P* = .019). As shown in Figure 2B, the fifth year after HGD resection was the peak period of MGC (48-60 months: 5.88%), while the third year after EGC resection was the peak period (24-36 months: 4.55%).

### Risk Factors for Metachronous Gastric Cancer Occurrence

To identify population susceptible to recurrence, we analyzed the baseline characteristics of patients. Age over 60 years (87.5% vs. 65.3%, *P* = .026), persistent* H. pylori *infection (54.2% vs. 25.6%, *P* = .012), background mucosal atrophy (50% vs. 25.2%, *P* = .009), and intestinal metaplasia (70.5% vs. 40.5%,* P* = .001) were more frequently observed in MGC patients (Supplementary Table 1). There were no significant differences in sex (*P* = .678), family history of cancer (*P* > .999), lesion location (*P* = .814), macroscopic type (*P* = .432), synchronous lesions (*P* = .600), and histopathology of primary cancer (*P* = .475). Multivariate Cox regression analysis revealed that age over 60 years (HR = 4.348, 95% CI = 1.290-14.659, *P* = .018), intestinal metaplasia (HR = 3.226, 95% CI = 1.132-9.193, *P* = .028), and persistent* H. pylori *infection (HR = 3.002, 95% CI = 1.177-7.659, *P* = .021) were independent risk factors for MGC occurrence (Table 1).

### Clinicopathological Characteristics of Metachronous Gastric Cancer

**Figure 3.**
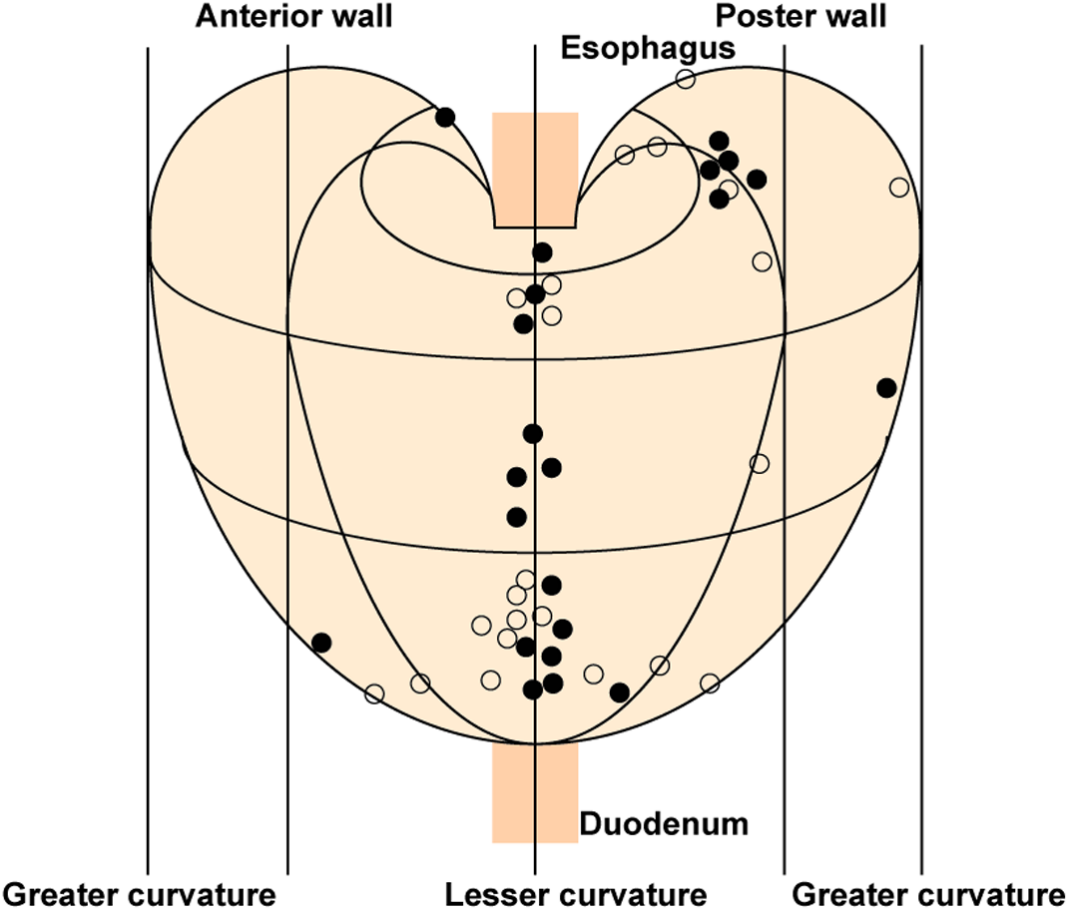
Schematic diagram of the location distribution of metachronous and primary lesions. Two cases were excluded for lack of a detailed pathological evaluation of metachronous lesions. Closed circle, the metachronous lesion; open circle, the primary lesion.

To acquire in-depth knowledge of MGC, we paid attention to the endoscopic and pathological characteristics of metachronous lesions (Supplementary Table 2). Two patients without surgery or endoscopic resection were excluded for lack of detailed pathological evaluation of metachronous lesions. The median duration before the MGC occurrence was 31 months (range, 12 to 88 months). Most metachronous lesions were smaller than 20 mm, except for 1 differentiated tumor of 24 mm. Nearly half of the metachronous lesions (12/22, 55%) occurred in the same third of the stomach as the primary lesions and 59% (13/22) occurred in the lesser curvatures (Figure 3). Correlation analysis revealed that the cross-sectional location of metachronous lesions was significantly correlated with that of primary lesions [Contingency coefficient (C) = 0.627, *P* = .027; Table 2]. When the primary lesions were located in the posterior walls, the metachronous lesions were prone to develop in the lesser curvatures (7/7, 100%; C = 0.494, *P* = .008), and primary lesions in the lesser curvatures predisposed metachronous lesions to developing in the posterior walls (5/10, 50%; C = 0.422, *P* = .029; Table 3). The macroscopic types (C = 0.457, *P* = .214) and histopathology (C = 0.326, *P* = .625) of metachronous lesions were not similar to those of primary lesions.

### Treatment and Prognosis of Metachronous Gastric Cancer

**Figure 4.**
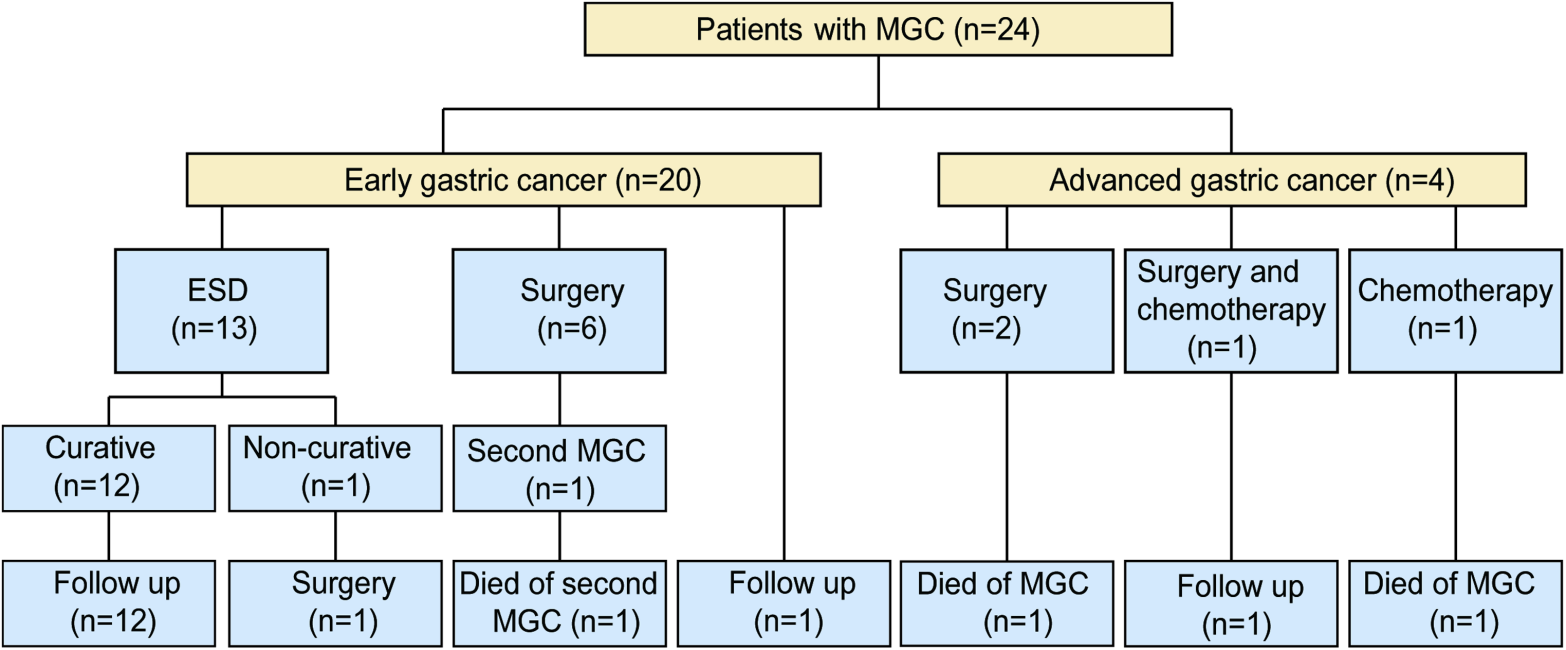
Treatments and outcomes of patients with MGC. ESD, endoscopic submucosal dissection; MGC, metachronous gastric cancer.

Among 24 MGC patients, 20 patients (20/24, 83.3%) developed EGC (Figure 4). Repeated ESD was the most common treatment, which was performed for 13 patients (13/20, 65%) with a curative resection rate of 92.3%. Six patients (6/20, 30%) received additional radical gastrectomy, 1 of whom experienced a second MGC and died 4 years later. The remaining patient chose regular surveillance rather than treatment due to old age and poor physical condition. Four patients (4/24, 16.7%) developed advanced gastric cancer, 1 of whom died after surgical resection and 1 died during chemotherapy.

## DISCUSSION

The MGC is a major challenge for endoscopic resection of gastric cancer, and the follow-up strategy after ESD remains vague. This study systematically analyzed the recurrence patterns of MGC after ESD and first found its temporal-spatial association with the primary tumor.

In our study, the overall incidence of MGC was 8.4% and the 5-year cumulative incidence was 13.4%. The result was consistent with studies previously published.^3,4^ Clarifying the recurrence tendency of MGC contributes to specifying the necessary duration and interval of endoscopic follow-up. Multicenter cohort studies found a nearly linear increase in the cumulative incidence of MGC, implying an ongoing increasing recurrence risk with time after endoscopy resection.^5,15^ Min et al^16^ proposed that annual or biannual surveillance endoscopy should be performed for at least 5 years after curative ESD,^16^ which was supported by our data. In this study, more than half of MGCs developed in the second or third year after ESD, and nearly 90% were detected within 5 years. However, long-term endoscopic follow-up (>5 years) has rarely been mentioned in most studies. In our data, sporadic cases also occurred 5 years after ESD (the longest interval was 88 months), suggesting that endoscopic surveillance cannot be discontinued even after 5 years.

Given the differences between primary EGC and HGD in pathogenicity and invasiveness, we stratified the incidence of MGC according to the histopathology of primary tumors. The average time to MGC after EGC resection has been reported to be 3.1 years,^17^ while that after HGD resection remains unclear. In our results, the peak period of MGC in patients with primary HGD was the fifth year, relatively later than that in patients with primary EGC, implying the predilection period of MGC was associated with the histopathology of the primary lesions. This may be because gastric mucosa harboring severe lesions is thought to accumulate more carcinogenic factors promoting tumor progressions, such as multiple precancerous lesions and aberrant DNA methylation.^18,19^ In this regard, we speculate that patients with low-grade dysplasia might take a longer time to develop metachronous cancers after ESD. More specifically, the incidence of MGC was comparably lower in studies including intraepithelial neoplasia during the limited follow-up period.^9,20^ Instead of simple numerical differences, our study included the follow-up duration and population distribution in the analysis to minimize patient selection bias, reflecting the epidemiological trends of MGC. This is the strength of our study that distinguishes it from previous studies. This finding revealed different predilection periods of MGC after EGC and HGD resection, which provides crucial clues to individualized endoscopic follow-up strategies. For example, more frequent endoscopic examinations may be recommended during the predilection periods of MGC. And long-term endoscopy surveillance of more than 5 or even 10 years is encouraged, especially for patients with primary HGD or low-grade dysplasia resection.

Another strength of our study is to present new insights into the characteristics of metachronous lesions. In multiple gastric cancer, clinicopathologic similarities of the main and minor lesions of synchronous early gastric cancer have been demonstrated,^21^ while associations of the metachronous lesions with the primary lesions remain unknown. In the present study, half of the metachronous lesions occurred in the same third of the stomach as the primary lesions, and 59% developed in the lesser curvatures, which was concordant with previous reports.^22^ Notably, there was a significant correlation between the metachronous and primary lesions in the cross-sectional location, especially in the lesser curvature and posterior wall. The location of EGCs showed that the lesser curvature and posterior wall were the common sites of involvement.^23^ Intestinal-type gastric cancer is preceded by a cascade of precancerous lesions, and atrophic mucosal changes frequently advance along the lesser curvature and extend to the posterior walls.^24,25^ The phenomenon that metachronous lesions often occur adjacent to the primary lesions was parallel to the “collision tumor theory.”^21^ Our study revealed a previously unappreciated locational correlation between the metachronous and primary lesions, which contributes to identifying MGC. Endoscopists could investigate the potential recurrence sites more intensively according to the locations of primary lesions in case of missing minimal lesions.

Nevertheless, there was no similarity between the macroscopic and histopathological types of the metachronous and primary lesions, which may be related to microsatellite instability (MSI). The MSI was more frequently detected in patients with MGC.^26^ Despite metachronous cancers occurring in the same stomach in different years, genetic alterations of these cancers were completely different, similar to the heterogeneity associated with MSI.^27^ The biological characteristics of cancer tend to be based on molecular phenotype, and characteristic diversities of MGC may be attributed to the heterogeneity of MSI. Since the further study is required on the role of molecular mechanisms in clinicopathological characteristics, our data could encourage a focus on the molecular biology of MGC.

Several limitations of this study cannot be ignored. First, this was a single-centered retrospective study. Second, we excluded a few patients based on strict criteria. The small sample size might result in selection bias, although we consider the sample size adequate to support the findings of this study. Third, we speculated that the characteristic diversities of metachronous lesions were related to genetic alterations, such as MSI, but definitive molecular phenotypes were not confirmed in the present study. The potential mechanisms of metachronous recurrence require further study. Despite these limitations, to our knowledge, this study is the first on the temporal-spatial association between MGCs and primary tumors, which has clinical implications for endoscopic surveillance after ESD. Further prospective large-scale studies are warranted to validate our preliminary results.

## CONCLUSION

In conclusion, we studied the recurrence patterns of MGC after ESD and evaluated the associations between the metachronous and primary lesions. We found the predilection period of MGC after HGD resection was relatively later than that after EGC resection. The metachronous and primary lesions shared close locations, but not similar pathological characteristics. Individualized endoscopic surveillance should be carried out meticulously, taking into account the characteristics of primary lesions.

## Figures and Tables

**Table 1.** Cox Proportional Analysis of Risk Factors for the Development of MGC

	Univariate Analysis		Multivariate Analysis	
	HR (95% CI)	*P*	HR (95% CI)	*P*
Sex, male	1.132 (0.713-1.798)	.599		
Age, ≥60 years	3.902 (1.163-13.095)	.028	4.348 (1.290-14.659)	.018
Smoking	1.804 (0.768-4.241)	.176		
Drinking	0.924 (0.314-2.716)	.886		
Family history of cancer	1.048 (0.357-3.079)	.932		
Synchronous lesions	2.273 (0.533-9.698)	.267		
Gastric atrophy	2.710 (1.216-6.041)	.015	1.265 (0.516-3.106)	.607
Intestinal metaplasia	4.133 (1.640-10.417)	.003	3.226 (1.132-9.193)	.028
*H. pylori *infection status				
Negative group	1			
Eradication group	1.224 (0.357-4.194)	.748		
Persistent infection group	3.822 (1.521-9.608)	.004	3.002 (1.177-7.659)	.021
Histopathology of primary tumor, EGC	1.620 (0.724-3.627)	.24		

EGC, early gastric cancer; HR, hazard ratio; MGC, metachronous gastric cancer.

**Table 2.** Correlation Analysis Between the Metachronous and Primary Lesions^†^

Primary Lesion	Metachronous Lesion			*P*
Longitudinal location	Upper third	Middle third	Lower third	.187
Upper third	6 (66.7%)	2 (22.2%)	1 (11.1%)	
Middle third	0 (0)	0 (0)	1 (100%)	
Lower third	3 (25%)	3 (25%)	6 (50%)	
Cross-sectional location	Greater curvature	Lesser curvature	Posterior wall	.027
Greater curvature	1 (25%)	2 (50%)	1 (25%)	
Lesser curvature	1 (10%)	4 (40%)	5 (50%)	
Anterior wall	1 (100%)	0 (0)	0 (0)	
Posterior wall	0 (0)	7 (100%)	0 (0)	
Macroscopic type	Elevated	Flat	Depressed	.214
Elevated	2 (28.6%)	2 (28.6%)	3 (42.9%)	
Flat	0 (0)	5 (71.4%)	2 (28.6%)	
Depressed	0 (0)	5 (62.5%)	3 (37.5%)	
Histopathology^‡^	Well differentiated	Moderately differentiated	Poorly differentiated	0.625
HGD	8 (72.7%)	2 (18.2)	1 (9.1%)	
Well differentiated	3 (42.9%)	3 (42.9%)	1 (14.3%)	
Moderately differentiated	2 (50%)	2 (50%)	0 (0)	

HGD, high-grade dysplasia.

^†^Two patients were excluded for lack of detailed pathological evaluation of metachronous lesions.

^‡^Tumor histological types were determined according to the World Health Organization classification.

**Table 3.** Correlation Analysis Between the Metachronous and Primary Lesions in the Cross-Sectional Location

Primary Lesion	Metachronous Lesion		*P*
	Lesser curvature	Other	.008
Posterior wall	7 (100%)	0 (0)	
Other	6 (40%)	9 (60%)	
	Posterior wall	Other	.029
Lesser curvature	5 (50%)	5 (50%)	
Other	1 (18.3%)	11 (91.7%)	

**Supplementary Table 1.** Baseline Characteristics of the Patients

	**Patients without MGC** **(n = 262)**	**Patients with MGC** **(n = 24)**	***P***
**Sex, male**	186 (71.0%)	18 (75.0%)	.678
**Age,** **≥ 60 years**	171 (65.3%)	21 (87.5%)	.026
**Smoking**	62 (23.7%)	8 (33.3%)	.292
**Drinking**	53 (20.2%)	4 (16.7%)	.880
**Family history of cancer**	47 (17.9%)	4 (16.7%)	>.999
***H. pylori*** **infection status**			.012
Negative group	127 (48.5%)	7 (29.2%)	
Eradication group	68 (26.0%)	4 (16.7%)	
Persistent infection group	67 (25.6%)	13 (54.2%)	
**Synchronous lesions**	10 (3.8%)	2 (8.3%)	.600
** Atrophy**	66 (25.2%)	12 ()	.009
** Intestinal metaplasia**	106 (40.5%)	18 (75%)	.001
**Longitudinal location**			.814
Upper third	88 (33.6%)	9 (37.5%)	
Middle third	16 (6.1%)	2 (8.3%)	
Lower third	158 (60.3%)	13 (54.2%)	
**Macroscopic type**			.432
Elevated	102 (38.9%)	8 (33.3%)	
Flat	103 (39.3%)	8 (33.3%)	
Depressed	57 (21.8%)	8 (33.3%	
**Histopathology of primary tumor**			.475
EGC	122 (46.6%)	13 (54.2%)	
HGD	140 (53.4%)	11 (45.8%)	

Data were expressed as n (%). The groups were compared with Chi-square test or Fisher’s exact test. MGC, metachronous gastric cancer; EGC, early gastric cancer; HGD, high-grade dysplasia.

**Supplementary Table 2.** Clinicopathological Characteristics of MGC Lesions^†^

	**Total (n = 22)**
**Lesion size, mm, mean ± SD**	12.7 ± 6.1
**Location**	
Upper third	9
Middle third	1
Lower third	12
**Macroscopic type**	
Elevated	7
Flat	7
Depressed	8
**Histopathology**	
Well differentiated	13
Moderately differentiated	7
Poorly differentiated	2
**Invasion depth**	
Mucosa	19
Submucosa or muscularis propria	3
** Lymphovascular invasion**	2
**Disease-free duration, months, median (range)**	31 (12-88)

^†^Two patients were excluded for lack of a detailed pathological evaluation of metachronous lesions.
